# Identification of Long Non-coding and Messenger RNAs Differentially Expressed Between Primary and Metastatic Melanoma

**DOI:** 10.3389/fgene.2019.00292

**Published:** 2019-04-05

**Authors:** Ledong Sun, Zhiguang Guan, Shanshan Wei, Rui Tan, Pengfei Li, Lu Yan

**Affiliations:** ^1^Department of Dermatology, Zhujiang Hospital, Southern Medical University, Guangzhou, China; ^2^Department of Plastic Surgery and Dermatology, Taishan People’s Hospital, Tangshan, China

**Keywords:** melanoma, long non-coding RNA, messenger RNA, expression profile, tumorigenesis

## Abstract

**Purpose:** Melanoma is the most aggressive and life-threatening cutaneous cancer. To explore new treatment strategies, it is essential to identify the mechanisms underlying melanoma tumorigenesis and metastasis.

**Methods:** In the current study, we demonstrated altered expression of long non-coding RNA (lncRNA) and messenger RNA (mRNA) in melanoma using data from the Cancer Genome Atlas (TCGA) database. Gene Ontology (GO), Kyoto Encyclopedia of Genes and Genomes (KEGG) enrichment, and protein–protein interaction (PPI) analyses were conducted. We also constructed a functional lncRNA-mRNA regulatory network and Kaplan-Meier analysis.

**Results:** We identified 246 differentially expressed (DE) lncRNAs and 856 DEmRNAs. A total of 184 DElncRNAs and 428 DEmRNAs were upregulated in metastatic melanoma, while all others were downregulated. Additionally, we investigated the co-expression pattern of 363 genes, among which 26 upregulated lncRNAs, 9 down- regulated lncRNAs, 49 upregulated mRNAs and 151 downregulated mRNAs were identified as being co-expressed with others. Survival analysis suggested high levels of 14 lncRNAs and 10 mRNAs may significantly increase or decrease overall survival. These differentially expressed genes are also potentially prognostic in melanoma.

**Conclusion:** Our findings observe potential roles for lncRNAs and mRNAs during melanoma progression and provide candidate biomarkers for further studies.

## Introduction

Cutaneous melanoma is the most aggressive type of skin cancer, and melanoma metastasis accounts for the vast majority of patient deaths from skin cancer. For early stage melanoma, surgical excision is used as the standard approach to remove localized lesions and results in a good prognosis. However, the prognosis of patients with metastatic melanoma is extremely poor. The 5-year survival rate of metastatic melanoma is only 5–10%, while that of non-metastatic melanoma is estimated to be approximately 90%. Although immunotherapy and molecularly targeted drugs are emerging as attractive new therapeutic approaches (Reddy et al., 2016), their utility is restricted to only a portion of patients who often acquire drug resistance, and the therapeutic options against late-stage melanoma remain very limited. A comprehensive understanding of the genetic and epigenetic underpinnings of melanoma initiation and progression would facilitate the identification of new biomarkers and therapeutic targets for metastatic melanoma.

Recently, actively transcribed lncRNAs have been demonstrated to be involved in complex cancer genome regulatory networks rather than representing merely extraneous transcription as originally believed. lncRNAs are defined as endogenous cellular RNA transcripts longer than 200 nucleotides without protein-coding capacity. Their structures resemble mRNAs, but they exhibit lower levels of expression and more tissue-specific expression patterns than protein-coding genes (Cabili et al., 2011). lncRNAs play extensive functions encompassing biological functions including biological evolution, cell proliferation, morphogenesis, cell cycle, immune and inflammatory response, and restrainting miRNA, mRNA, and proteins from combining with their intended targets (Cathcart et al., 2015; Chen et al., 2017; Yu et al., 2018). The expression of lncRNAs varies significantly (Yan et al., 2015) in different tissues and stages of development.

To date, the roles of most lncRNAs have not been elucidated in spite of their relatively huge representation in genomes. It is well known that co-expressed genes are more likely to be co-regulated and functionally related (Stuart et al., 2003). Thus, identifying co-expressed protein-coding genes may reveal functions to associated lncRNAs through use of publicly available genome-wide datasets and computational methods (Signal et al., 2016).

In recent years, TCGA^1^ has generated comprehensive, multi-dimensional maps of the crucial genomic changes in numerous cancers, which help to provide an understanding of how such changes interact to drive diseases. Weighted correlation network analysis (WGCNA), a powerful correlation network method, is used to reconstruct gene co-expression network relationships between differentially expressed lncRNAs (DElncRNAs) and differentially expressed mRNAs (DEmRNAs). Gene Ontology Enrichment Analysis Software Toolkit (GOEAST), PANTHER, and the database for annotation, visualization, and integrated discovery (DAVID) (Huang da et al., 2009) combined with the Search Tool for the Retrieval of Interacting Genes (STRING) database^2^ are commonly used to explore the biological functions of the genomic changes. This method has been successfully applied in various biological contexts to identify candidate biomarkers or therapeutic targets, e.g., in cancer, mouse genetics, yeast genetics, and the analysis of brain imaging data. In the present study, we used a computational strategy to perform a systematic study of lncRNAs significantly altered in the transition from primary to metastatic melanoma, and constructed a functional lncRNA-mRNA regulatory network aiming to contribute to a comprehensive understanding of the differentially expressed genes (DEGs) in melanoma and their function. We identified novel melanoma-associated lncRNAs and predicted their potential pathological and biological roles in cancer development and gene regulation. This work may provide some powerful evidence to guide subsequent experimental studies on the altered genes in melanoma as candidate targets for treatment development or as biomarkers.

## Materials and Methods

### Differentially Expressed lncRNA and mRNA Analyses

Transcriptome sequencing data by RNA-seq were downloaded from the publically available TCGA database, including 103 primary melanoma frozen tissue and 368 metastatic melanoma tissue datasets. All samples were collected from individuals of different races and genders. This dataset comprised of called gene counts for 57,073 mRNAs and non-coding (nc)RNAs. To obtain genome-wide lncRNA and mRNA expression profiles, normalized expression data were subsequently analyzed for DElncRNAs and mRNAs using Bioconductor package (limma, version 3.4.0) in R software (version 3.4.0) with default parameters (Ritchie et al., 2015). A total of 246 DElncRNAs and 856 DEmRNAs remained in the differential expression analysis using “edgeR” in R. Statistical significance was defined as |log_2_foldchange| >2 and *P-*value < 0.01. The FDR method by the Benjamini and Hochberg method was applied to correct for multiple testings. Hierarchical clustering, shown in the format of a heatmap and a volcano plot, of the DElncRNA and DEmRNA expression profiles was performed by using ”RColorBrewer,” “pheatmap,” and “plot” in R language.

### Co-expression Network Construction of the lncRNA-mRNA Regulatory Network

A DElncRNA-DEmRNA co-expression network was generated to explore the key roles of DElncRNAs and DEmRNAs in the progression of melanoma. As a systems biology method, gene co-expression network analysis was performed by the WGCNA package (Langfelder and Horvath, 2008) to construct a co-expression network between the onco-lncRNAs and their target mRNAs, based on the signed Pearson Correlation Coefficient between their normalized expression levels as provided by Cuffnorm (Trapnell et al., 2012). As to achieve a scale-free topology, we set β = 7 and modified the pairwise correlation into an adjacency matrix of connection strengths through a soft-thresholding approach. Only edges with weight above a threshold of 0.8 are displayed. In the co-expression network, nodes represented DEGs, and the correlation of gene expression pattern was defined as connectivity degree among genes (Ghazalpour et al., 2006). The network was visualized using Cytoscape 3.5.1.

### Bioinformatics and PPI Network Analysis

Gene Ontology analysis is a functional analysis for exploring DEmRNAs with GO categories. The predicted target genes above were input into David^3^, GOEAST^4^ and PANTHER^5^, which utilized GO to identify coexisting biological process, cellular component, and molecular function represented in the gene profile. Furthermore, we also used the DAVID database to analyze the potential functions of these genes in the pathways (*P* < 0.05). The results were in accordance with those from KEGG^6^.

Protein–protein interaction information of DEmRNAs was acquired from the STRING database^7^. Edges with weight above a threshold of 0.4 are displayed. Hub genes were found by using “barplot” in R language.

### Survival Rate Analysis

Furthermore, we investigated whether the co-expressed DElncRNAs-DEmRNAs were correlated with potential good or poor overall survival (OS). Clinical data from TCGA with survival time were collected. The gene expression was labeled as high or low using a dichotomy method (Tian et al., 2017). OS rate analysis was performed by using “hash” and “survival” in R to examine the association between the gene expression levels and the overall lifetime (Gentleman et al., 2006). Differences in the OS between the two groups were estimated and compared by the Kaplan–Meier method with a log-rank test. *P*-values less than 0.05 were considered significantly different.

### Receiver Operating Characteristic (ROC) Analyses

In order to evaluate the diagnostic value of candidate DElncRNAs, ROC analyses were performed using the “survivalROC” package in R language. AUC under a binomial exact confidence interval was calculated to generate the ROC curve. Univariate Cox and multivariate Cox analyses were evaluated separately.

## Results

### Identification of DElncRNAs and DEmRNAs

**FIGURE 1:**
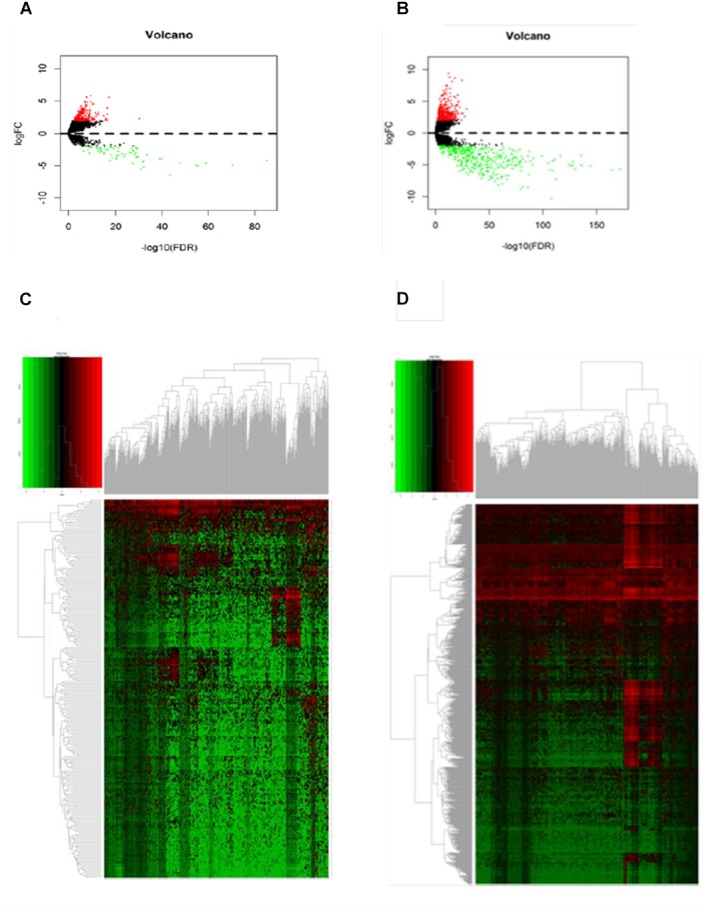
Differentially expressed genes between primary and metastatic melanoma (fold change > 2, FDR < 0.01). **(A)** Volcano plot of the FDR as a function of weighted fold change for DElncRNAs and **(B)** DEmRNAs, red dots represent significantly upregulated expressed genes and green dots represent significantly downregulated expressed genes (fold change > 2, FDR < 0.01). **(C)** Heat map for potential lncRNAs (*n* = 246) showed significant expression changes, in which 62 were downregulated and 184 were upregulated. Red through green color indicates high to low expression level. **(D)** Heat map for potential mRNAs showed significant expression changes (*n* = 856), in which 428 were downregulated and 428 were upregulated.

A gene expression dataset generated by RNA sequencing (RNA-Seq) was downloaded from TCGA. The dataset included the expression levels detected for 32,068 different probes on 103 primary melanoma frozen tissues and 368 metastatic melanoma tissues. Among these, 246 DElncRNAs and 856 DEmRNAs were identified (log2fold change > 2, *FDR* < 0.01) by an analysis in R language. We noted 184 DElncRNAs and 428 DEmRNAs were upregulated in metastatic melanoma, with 62 DElncRNAs and 428 DEmRNAs being downregulated, which were described as a volcano plot (Figure 1A,B).

Hierarchical clustering of the DElncRNA and DEmRNA expression profiles was performed by using “pheatmap” in R language. Heatmaps of the differential expression of the genes allowed for easy discrimination between primary and metastatic melanoma (Figure 1C,D). The top 20 significantly upregulated DElncRNAs are shown in Table 1 and downregulated DElncRNAs in Table 2.

**Table 1:** The top 20 upregulated DElncRNAs in metastatic melanoma (*p* < 0.01).

GenBank Accession
number	Chromosome/location	Ensembl gene ID	Gene type	log2 Fold Change	*FDR*
LINC01235	9p23	ENSG00000270547	LincRNA	2.35954048	6.73E-31
LINC00824	8q24.21	ENSG00000254275	Processed_transcript	5.538087262	7.46E-18
AC243960.1	19, CH17-20A21	ENSG00000268027	LincRNA	2.163950636	3.65E-17
MEOX2-AS1	7p21.2 1q43	ENSG00000229108	LincRNA	3.850613425	4.59E-17
CHRM3-AS2	1q43	ENSG00000233355	Antisense	3.053111642	4.63E-17
LINC00861	8q24.13	ENSG00000245164	LincRNA	2.355695169	1.76E-16
AL365361.1	1, clone RP11-284N8	ENSG00000259834	LincRNA	2.060003316	1.92E-16
LINC01624	6q27	ENSG00000227508	LincRNA	2.088952578	2.18E-16
LINC01215	3q13.12	ENSG00000271856	LincRNA	2.792953673	1.38E-15
LINC01857	2q33.3	ENSG00000224137	LincRNA	2.056385031	8.76E-14
AC083967.1	8, Clone: RP11-865I6	ENSG00000254337	LincRNA	3.368320439	2.92E-13
AC023301.1	18,Clone: RP11-713C5	ENSG00000265579	Sense_intronic	3.060224388	3.51E-13
LINC01781	1p31.1	ENSG00000234184	LincRNA	2.982915086	4.19E-13
AC091806.1	X, clone RP11-320G24	ENSG00000236393	LincRNA	3.035227377	1.92E-12
LINC00402	13, NR_144451.1	ENSG00000235532	LincRNA	2.710541667	3.03E-12
AC106882.1	4, clone RP11-768B22	ENSG00000248571	Antisense	2.690704212	6.64E-12
AL354719.2	6, clone RP11-59D5	ENSG00000236345	Antisense	3.297587187	6.73E-12
LINC02422	12p11.21	ENSG00000255760	LincRNA	2.195008576	1.32E-11
AC133961.1	UNK, clone RP13-494C23	ENSG00000251009	LincRNA	2.275195921	2.17E-11
AC104051.2	8, clone CTD-2339F6	ENSG00000254139	LincRNA	4.511208671	4.41E-11

**Table 2:** The top 20 downregulated DElncRNAs in metastatic melanoma (*p* < 0.01).

GenBank Accession
number	Chromosome/location	Ensembl gene ID	Gene type	log2 Fold Change	*FDR*
AL512274.1	11p15.5	ENSG00000261068	LincRNA	-4.180138414	6.63E-86
AC012313.4	7p15.2	ENSG00000268307	LincRNA	-4.883495525	4.02E-71
FAM83A-AS1	20q13.33	ENSG00000204949	Antisense	-4.559483782	1.07E-59
LINC01214	10q23.2	ENSG00000243550	LincRNA	-5.03190323	1.83E-56
FAM41C	20q13.33	ENSG00000230368	LincRNA	-4.980820757	1.37E-55
NCF4-AS1	14, clone C-2540L5	ENSG00000183822	Antisense	-4.634503046	2.78E-48
LINC01527	1q32.1	ENSG00000224308	Antisense	-6.526109341	3.95E-44
LINC02159	10q26.3	ENSG00000253417	LincRNA	-3.857391312	9.38E-41
C7orf71	16, clone RP11-161M6	ENSG00000222004	LincRNA	-3.992297835	7.77E-40
AC083841.1	17q25.1	ENSG00000253196	Antisense	-3.322042539	3.73E-34
AC022081.1	22q13.31	ENSG00000256513	LincRNA	-5.249157107	8.22E-33
AL512363.1	14q32.2	ENSG00000224984	Antisense	-4.383485567	1.38E-32
LINC00302	17, clone CTD-2008P7	ENSG00000176075	LincRNA	-5.562606502	1.63E-31
CALML3-AS1	17,Clone: CTD-2008P7	ENSG00000205488	Antisense	-3.568350471	3.29E-30
AC010503.4	6, clone RP1-153P14	ENSG00000275234	Antisense	-2.334423603	6.20E-30
GLIS3-AS1	12q12	ENSG00000237009	Antisense	-3.664848596	1.22E-29
LINC01343	16, clone RP11-473M20	ENSG00000237290	LincRNA	-2.960116345	1.90E-29
WFDC21P	19p13.12	ENSG00000261040	Processed_transcript	-2.160257549	1.90E-29
AL033384.1	1p21.3	ENSG00000236740	LincRNA	-3.256978817	3.46E-29
AC082651.3	7p15.2	ENSG00000243491	LincRNA	-3.327036008	7.88E-29

**FIGURE 2:**
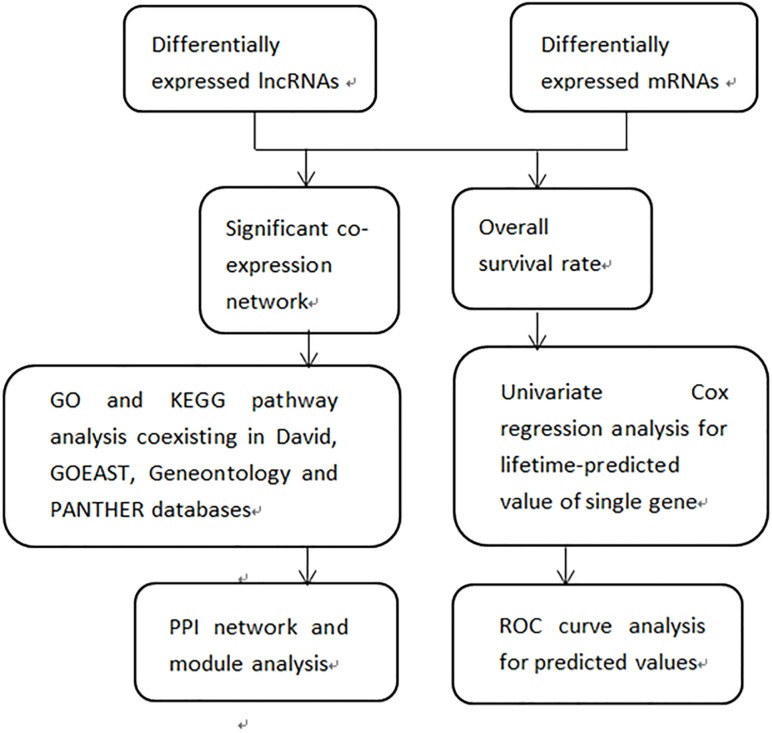
Flow diagram of the study design. DElncRNAs and DEmRNAs were investigated between 103 primary and 368 metastatic melanomas (log2fold change > 2, FDR < 0.01)

The flow diagram of our research design aiming to identify DEGs is shown in Figure 2. We created a gene co-expression network to screen out lncRNAs targeting mRNAs and hub genes among the co-expressed DEGs. Then we performed GO and KEGG analysis on DEGs, followed by analysis of the PPI network. Finally, we verified the prognostic value of DEG expression for patients with melanoma using the comprehensive survival analysis platform Kaplan-Meier plotter. We conducted univariate Cox regression analyses to determine lifetime-predicted values of single genes. We performed ROC curve analyses to obtain the predicted values of multivariate DEGs.

### Co-expression of lncRNAs and mRNAs in Melanoma

**FIGURE 3:**
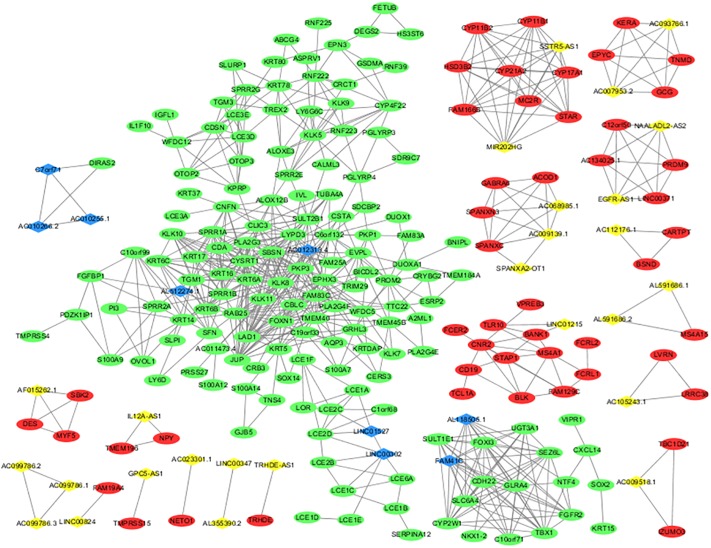
lncRNA and mRNA co-expression network in melanoma. The co-expression network established with 235 nodes and 762 edges, in which only edges with weight(w) above a threshold of 0.8 are displayed. The yellow nodes denote upregulated lncRNAs and the blue nodes denote downregulated lncRNAs. The red nodes denote upregulated mRNAs and the green nodes denote downregulated mRNAs.

A DElncRNA–DEmRNA co-expression network determined by WGCNA was used to reveal the roles and functional mechanisms of lncRNAs and their targeted mRNAs in melanoma (Terai et al., 2016). DElncRNAs and their significantly related mRNAs were imported to draw the network using Cytoscape (version 3.4.3). The co-expression network was composed of 235 network nodes and 762 connection edges, among which 26 upregulated lncRNAs, 9 downregulated lncRNAs, 49 upregulated mRNAs, and 151 downregulated mRNAs were identified to have interregulated relationships (Figure 3). Within the network, several single lncRNAs were co-expressed with multiple mRNAs such as AL51224.1 and AC012313.4, whereas individual mRNAs were targeted by multiple lncRNAs such as AC010266.2, C7orf71, and AC010255.1. Additionally, there were a few co-expression relationships between several lncRNAs or mRNAs, which indicated a highly complex regulatory relationship between lncRNAs and mRNAs in melanoma.

### GO Term Enrichment Analysis

Gene Ontology term enrichment analysis results varied according to GO classification and the expression change of DEmRNAs. GO term enrichment of 363 dysregulated DEGs in the co-expression network was also found among three databases David, GOEAST, GO, and PANTHER. With respect to biological process, the DEmRNAs were significantly enriched in “keratinocyte differentiation,” ”keratinization,” ”peptide cross-linking,” and “epidermis development.” For cellular component, the DEmRNAs were significantly enriched in “extracellular space,” “extracellular region,” “keratin filament,” “desmosome,” “cornified envelope,” “intermediate filament cytoskeleton,” and “intermediate filament.” With regard to molecular function, the DEmRNAs were significantly enriched in “steroid hydroxylase activity,” “structural molecule activity,” ”structural constituent of epidermis,” and “heme binding.” Additional details regarding GO enrichment analysis results are shown in Table 3.

**Table 3:** GO term enrichment of dysregulated DEGs in co-expression network coexisting in David, GOEAST, Gene Ontology, and PANTHER.

GO ID	Term	*P-*value	Genes
Biological process		
GO:0030216	Keratinocyte differentiation	2.28E-36	LOR, LCE3A, S100A7, LCE3D, SPRR2G, TP63, LCE1B, LCE1A, SPRR2E, CERS3, CDSN, SPRR2A, TGM1, TGM3, LCE2D, IVL, LCE2C, FOXN1, LCE2B, C1ORF68, EVPL, CRCT1, LCE1E, SPRR1A, KRT16, LCE1F, SPRR1B, LCE1C, LCE1D, CSTA, LCE3E
GO:0031424	Keratinization	2.80E-36	LOR, LCE3A, LCE3D, SPRR2G, LCE1B, LCE1A, SFN, SPRR2E, SPRR2A, TGM1, TGM3, LCE2D, IVL, LCE6A, LCE2C, LCE2B, EVPL, KRT17, LCE1E, SPRR1A, LCE1F, KRT16, SPRR1B, LCE1C, LCE1D, CNFN, LCE3E
GO:0018149	Peptide cross-linking	7.55E-32	LOR, LCE3A, LCE3D, SPRR2G, LCE1B, LCE1A, SPRR2E, SPRR2A, TGM1, TGM3, LCE2D, IVL, LCE2C, LCE2B, C1ORF68, EVPL, CRCT1, LCE1E, SPRR1A, LCE1F, LCE1C, SPRR1B, LCE1D, CSTA, LCE3E
GO:0008544	Epidermis development	8.93E-30	S100A7, LCE3D, SPRR2G, SPRR2E, CDSN, KRT5, SPRR2A, OVOL1, ZNF750, KRT83, KLK7, NTF4, KLK5, FOXN1, LCE2B, GRHL3, GJB5, GRHL2, C1ORF68, EVPL, KRT17, LCE1E, SPRR1A, KRT16, SPRR1B, LCE1C, KRT15, KRT14
Cellular component		
GO:0005615	Extracellular space	2.62E-11	SLURP1, LYPD3, PRH1, KERA, S100A8, PRH2, S100A9, SCGB1A1, KRT33A, KRT33B, MS4A1, KLK11, KRT83, APCS, C10ORF99, NAPSA, PIGR, C8A, TACSTD2, CA6, CARTPT, SLPI, CSTA, SERPINB3, PLA2G3, EPYC, AMY1B, SEZ6, AMY1A, TG, BPIFB1, BPIFB2, VPREB3, SERPINA12, TAC1, SFTPA1, SFN, ZG16B, ALB, FGB, APOC3, SERPINB13, FGFBP1, KLK7, LPO, KLK8, IL1F10, FETUB, KLK5, A2ML1, GCG, ORM1, AFM, NPY, CXCL14, SFTPA2, KRT78, IGFL1, MUC5AC
GO:0005576	Extracellular region	1.66E-06	SLURP1, S100A8, KERA, S100A7, S100A9, HTN3, HTN1, PGLYRP4, PGLYRP3, KLK10, BPIFA2, APCS, KRTDAP, C10ORF99, C8A, WFDC12, CA6, TUBA4A, PLA2G3, WFDC5, SMR3A, TG, FGFR2, BPIFB1, STATH, TAC1, SFTPA1, FAM19A4, NETO1, ALB, FGB, APOC3, SFTA2, CDA, HRG, FGFBP1, PRB2, PRB3, KLK7, NTF4, KLK9, FETUB, PRB4, EPHX3, A2ML1, S100A12, GCG, ORM1, AFM, NPY, CXCL14, SFTPA2, PRSS27, MUC5AC
GO:0045095	Keratin filament	4.98E-70	KRTAP4-4, KRTAP4-3, KRTAP4-2, KRTAP4-1, KRTAP5-3, KRTAP12-1, KRTAP12-3, KRT80, KRTAP12-2, KRTAP11-1, KRT83, KRTAP2-4, KRTAP2-2, KRTAP2-1, KRTAP10-3, KRTAP10-2, KRTAP10-5, KRTAP10-4, KRTAP10-7, KRTAP10-6, KRTAP10-9, KRTAP10-8, KRTAP5-5, KRTAP4-9, KRTAP3-1, KRTAP4-5, KRTAP4-6, KRTAP4-7, KRTAP3-2, KRT14, KRTAP4-8, KRTAP3-3, KRTAP1-1, KRTAP1-3, KRTAP1-5, KRTAP5-11, KRT6C, KRT6A, KRT6B, KRTAP16-1, KRTAP10-11, KRTAP10-12, KRTAP10-10, KRTAP10-1, KRT5, KRT4, KRTAP9-4, KRTAP9-6, KRTAP9-7, KRTAP9-3, KRTAP9-8, KRTAP9-9, KRTAP4-12, KRTAP4-11, KRT78
GO:0030057	Desmosome	2.27E-06	JUP, EVPL, PKP1, DSG3, PKP3, DSC2, CDSN
GO:0001533	Cornified envelope	8.56E-33	LOR, LCE3A, LCE3D, SPRR2G, LCE1B, LCE1A, SPRR2E, CDSN, SPRR2A, TGM1, LCE2D, IVL, LCE2C, LCE2B, C1ORF68, EVPL, CRCT1, LCE1E, SPRR1A, LCE1F, SPRR1B, LCE1C, LCE1D, CNFN, CSTA, LCE3E
GO:0045111	Intermediate filament cytoskeleton	0.057170437	DES, EVPL, TRIM29, KRT4
GO:0005882	Intermediate filament	7.28E-10	KRT6C, KRTAP8-1, KRT6A, KRT33A, KRT33B, JUP, KRT37, KRT80, DES, PKP1, KRT5, KRT17, KRT16, KRT15, KRT14, KRT4
Molecular function		
GO:0008395	Steroid hydroxylase activity	5.60E-04	CYP3A4, CYP21A2, CYP2C9, CYP11B2, CYP2W1
GO:0005198	Structural molecule activity	4.84E-36	KRT6C, LOR, KRT6A, LCE3A, LCE3D, SPRR2G, LCE1B, SPRR2E, LCE1A, KRT33A, KRT33B, KRT80, DES, KRTAP11-1, KRT5, FGB, SPRR2A, LCE2D, KRT4, IVL, KRT83, LAD1, LCE2C, LCE2B, C1ORF68, JUP, KRTAP3-1, KRT37, CRCT1, EVPL, LCE1E, KRT17, SPRR1A, KRT16, LCE1F, SPRR1B, KRT15, LCE1C, KRTAP3-2, KRT14, KRT78, KRTAP3-3, LCE1D, CSTA, LCE3E, SNTG2
GO:0030280	Structural constituent of epidermis	0.005857095	LOR, PKP1, KRTAP1-3
GO:0020037	Heme binding	1.01E-06	CYP3A4, LPO, CYP21A2, CYP2C9, CYP11B1, CYP11B2, DUOX1, CYP4F22, CYP2W1, CYP1A2, CYP17A1, HRG, IZUMO3

### KEGG Pathway Analysis

Through KEGG pathway analysis, we identified 12 significantly enriched pathways (Table 4): “Steroid hormone biosynthesis” (hsa00140), “Linoleic acid metabolism” (hsa00591), “Salivary secretion” (hsa04970), “Retinol metabolism” (hsa00830), “Aldosterone synthesis and secretion” (hsa04925), “Ovarian steroidogenesis” (hsa04913), “Arachidonic acid metabolism” (hsa00590), “Drug metabolism - cytochrome P450” (hsa00982), “Metabolism of xenobiotics by cytochrome P450” (hsa00980), “Chemical carcinogenesis” (hsa05204), “alpha-Linolenic acid metabolism” (hsa00592), and “Serotonergic synapse” (hsa04726). “Steroid hormone biosynthesis” was also revealed in the GO term analysis. The other results differed from the GO term enrichment analysis, implicating that fairly complicated molecular mechanisms exist in melanoma. Some pathways such as “Hematopoietic cell lineage” and “Ether lipid metabolism” were not significant (*P* > 0.05).

**Table 4:** KEGG pathway enrichment of dysregulated DEGs in melanoma (*p* < 0.05).

Items	Items_Details	*P*-value	Genes
hsa00140	Steroid hormone biosynthesis	2.72E-07	CYP3A4, HSD3B2, CYP17A1, CYP11B1, CYP21A2, CYP11B2, SULT2B1, SULT1E1, CYP1A2
hsa00591	Linoleic acid metabolism	1.75E-05	CYP3A4, CYP2C9, PLA2G4F, CYP1A2, PLA2G3, PLA2G4E
hsa04970	Salivary secretion	5.51E-05	PRB2, LPO, HTN1, PRH1, PRH2, CALML3, STATH, HTN3
hsa00830	Retinol metabolism	8.80E-04	CYP3A4, CYP2C9, ADH4, SDR16C5, ADH1A, CYP1A2
hsa04925	Aldosterone synthesis and secretion	0.0023636	HSD3B2, STAR, CALML3, CYP21A2, CYP11B2, MC2R
hsa04913	Ovarian steroidogenesis	0.002377024	HSD3B2, CYP17A1, STAR, PLA2G4F, PLA2G4E
hsa00590	Arachidonic acid metabolism	0.005587763	CYP2C9, ALOX12B, PLA2G4F, PLA2G3, PLA2G4E
hsa00982	Drug metabolism – cytochrome P450	0.007742837	CYP3A4, CYP2C9, ADH4, ADH1A, CYP1A2
hsa00980	Metabolism of xenobiotics by cytochrome P450	0.01038469	CYP3A4, CYP2C9, ADH4, ADH1A, CYP1A2
hsa05204	Chemical carcinogenesis	0.013551374	CYP3A4, CYP2C9, ADH4, ADH1A, CYP1A2
hsa00592	alpha-Linolenic acid metabolism	0.033433436	PLA2G4F, PLA2G3, PLA2G4E
hsa04726	Serotonergic synapse	0.039298908	CYP2C9, SLC6A4, ALOX12B, PLA2G4F, PLA2G4E

### PPI Network Analysis

**FIGURE 4:**
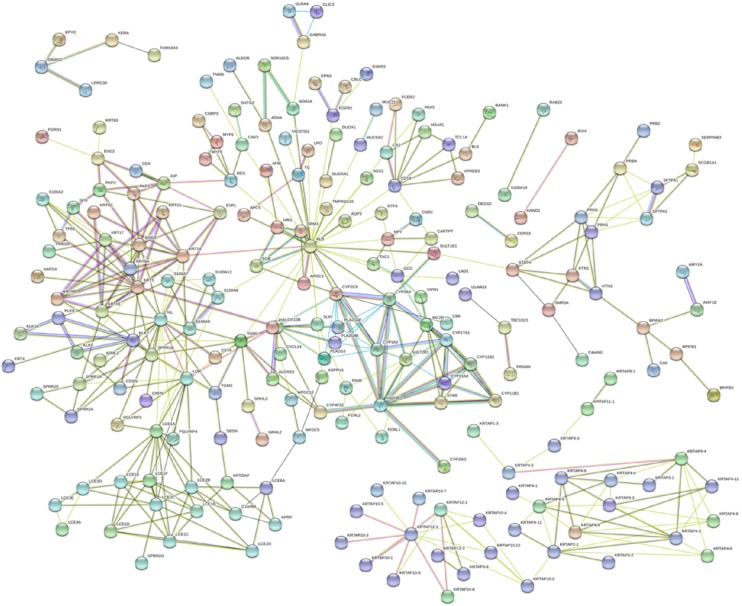
The PPI network constructed by the website STRING (https://string-db.org/). A total of 322 proteins are presented with color nodes; interactions are represented with edges. Only edges with weight (w) above a threshold of 0.4 are displayed.

To predict additional complex relationships, a PPI network of DEmRNAs, consisting of 327 nodes and 456 edges, was constructed using the STRING database and Cytoscape software. The top 18 DEmRNAs with a high degree of connectivity were selected as hub genes for melanoma. The hub genes comprised FGA, FGG, FGFR2, SOX2, S100A9, KRT5, CYP17A1, CYP21A2, FGF1, HSD3B2, FGB, NANOG, FGF10, S100A8, KRT14, FGFBP1, CYP11B2, and CYP11B1. Among these, FGA, FGB and FGG, FGFR2 and FGF1, SOX2 and NANOG, FGFR2 and FGF10, and S100A9 and S100A8 showed the highest node connection degree (Figure 4).

### Correlation of DElncRNAs and DEmRNAs With Overall Survival Rate

**FIGURE 5:**
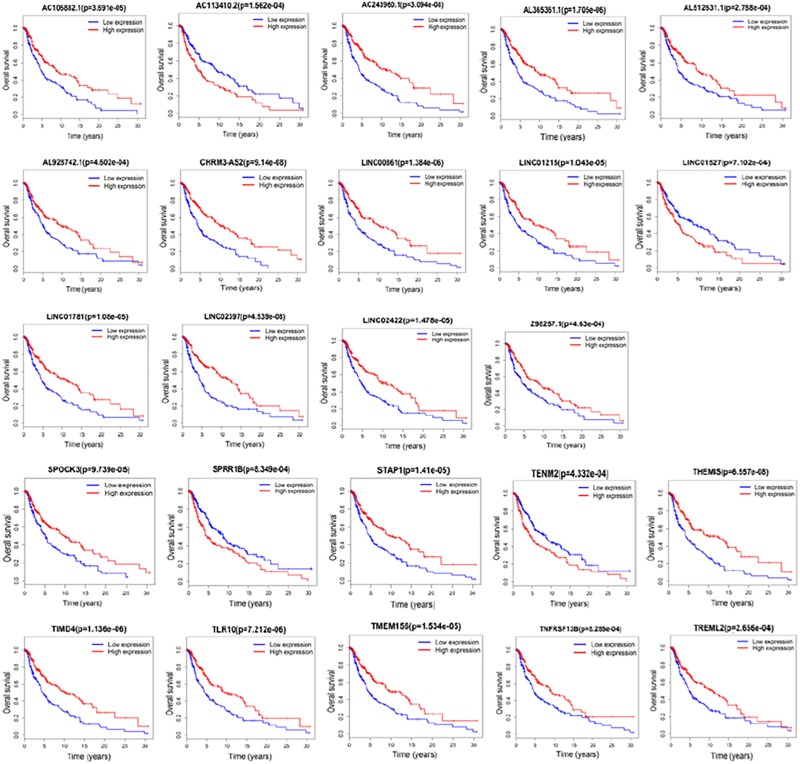
Kaplan–Meier analysis for overall survival rate of patients from TCGA. Log-rank test was performed to evaluate the survival differences between the two curves (*p* < 0.001). Overall survival rate for 14 lncRNAs and 10 mRNAs were identified significant.

We next examined whether the DElncRNAs and DEmRNAs were associated with the clinical outcome of patients with melanoma. We performed a univariate Cox regression analysis according to the survival data from TCGA. We performed a univariate Cox regression analysis according to the survival data from TCGA. We found that 14 lncRNAs and 10 mRNAs were significantly associated with patient survival (*P* < 0.001). Increased expression levels of 12 lncRNAs including AC106882.1, AL365361.1, AC243960.1, AL512631.1, AL928742.1, CHRM3-AS2, LINC00861, LINC01215, LINC01781, LINC02397, LINC02422, and Z98257.1 were correlated with higher survival rate. AC113410.2 and LINC01527 correlated with adverse prognosis. Furthermore, 8 mRNAs SPOCK3, STAP1, THEMIS, TIMD4, TLR10, TMEM156, TNFRSF13B, and TREML2 were associated with favorable outcome in patients with melanoma. Our results suggest that SPRR1B and TENM2, may act as risk factors and independent prognostic indicators. Detailed statistics of Kaplan–Meier survival curves are shown in Figure 5.

### Cox Regression Analysis and ROC Curve Analysis

To assess the prognostic value of the 246 candidate DElncRNAs and 856 DEmRNAs generated from TCGA database, we conducted Cox regression analysis and time-dependent ROC curve analyses along with calculations of the area under the curve (AUC). In univariate Cox analysis, 486 DEGs were found to have independent significance in predicting clinical outcome (*P* < 0.05). Among these, 243 DEGs were identified as having great significance (*P* < 0.001). The top 20 significant predicted-value DEGs are shown in Table 5. NCCRP1, FAM83C, A2ML1, GGT6, TRIM29, RHCG, SPRR2F, PKP1, RHOV, FETUB, KRT17, and CALML3 were identified as independent risk predictors while AL365361.1, AC243960.1, PLA2G2D, TIMD4, PTPRC, DNAJC5B, GPR174, and THEMIS were considered to be positive prognostic factors, which were in accordance with results from the survival rate analysis.

**Table 5:** Univariate Cox analysis for lifetime-predicted value of genes (TOP 20).

Gene	HR	*z*	*p*-value
AL365361.1	0.826354512	-5.909700466	3.43E-09
AC243960.1	0.831252414	-5.657993792	1.53E-08
NCCRP1	1.136040479	5.441268426	5.29E-08
FAM83C	1.128972106	5.394355953	6.88E-08
A2ML1	1.126045376	5.279541417	1.30E-07
PLA2G2D	0.905372153	-5.179133045	2.23E-07
GGT6	1.137579439	5.175315631	2.28E-07
TIMD4	0.858960901	-5.1104253	3.21E-07
PTPRC	0.862091666	-5.105751023	3.29E-07
TRIM29	1.100061145	5.097740391	3.44E-07
RHCG	1.126372087	5.081075698	3.75E-07
DNAJC5B	0.848528035	-5.064403499	4.10E-07
GPR174	0.862606849	-5.055840656	4.28E-07
SPRR2F	1.162350427	5.020615684	5.15E-07
PKP1	1.102784839	5.00654937	5.54E-07
RHOV	1.156982622	4.942331677	7.72E-07
FETUB	1.198131015	4.898590733	9.65E-07
KRT17	1.091777194	4.884241069	1.04E-06
CALML3	1.088087653	4.87205418	1.10E-06
THEMIS	0.878471787	-4.864231684	1.15E-06

**FIGURE 6:**
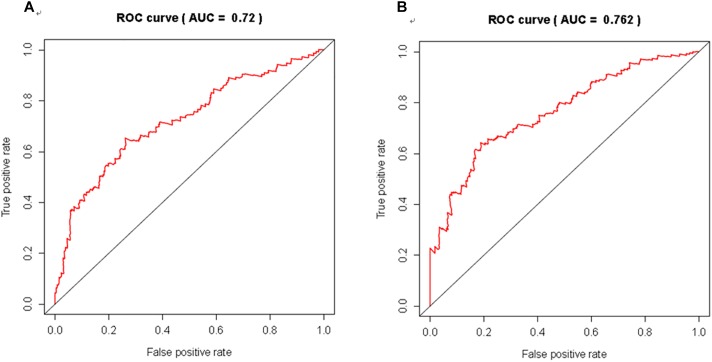
The discriminatory ability of the evaluated multivariate DEGs in melanoma was accessed with ROC curve. **(A)** for 3 year; **(B)** for 5 years.

We further evaluated the potential prognostic value of multivariates among the top 20 DEGs in melanoma using ROC curves. THEMIS, RHCG, PLA2G2D, NCCRP1, AL365361.1, and AC243960.1 were chosen as differences in the multivariate survival curve between high risk and low risk were significant (*P* = 7.02e-14) (Table 6). The AUC for 3 years was 0.72 (Figure 6A) and that for 5 years was 0.762 (Figure 6B).

**Table 6:** Multivariate Cox analysis for lifetime-predicted value of genes coxph(formula = Surv(futime, fustat) ∼ THEMIS + RHCG + PLA2G2D + NCCRP1 + AL365361.1 + AC243960.1, data = rt).

coef		exp(coef)	se(coef)	*z*	*p*
THEMIS	0.1355	1.1451	0.0619	2.19	0.02862
RHCG	0.0516	1.0529	0.0286	1.81	0.071
PLA2G2D	-0.0901	0.9138	0.0348	-2.59	0.00953
NCCRP1	0.1046	1.1103	0.0282	3.71	0.00021
AL365361.1	-0.1025	0.9026	0.0602	-1.7	0.08879
AC243960.1	-0.1168	0.8897	0.0763	-1.53	0.12577

*Likelihood ratio test = 73.7 on 6 df, p = 7.02e-14. n = 459, number of events = 218.*

## Discussion

Metastasis is a crucial factor in the determination of prognosis and survival of melanoma patients. Thus, elucidating the molecular mechanisms underlying melanoma metastasis is urgently needed. lncRNAs are now known to have crucial regulatory functions related to chromatin remodeling and gene expression in the progression of cancers (Wapinski and Chang, 2011). In the current study, a total of 246 DElncRNAs and 856 DEmRNAs were identified. We found 184 DElncRNAs were up-regulated whereas the remainder were down regulated. Furthermore, we investigated the co-expression pattern among 26 up-regulated lncRNAs,9 down-regulated lncRNAs, 49 up-regulated mRNAs and 151 down-regulated mRNAs. “Steroid hormone biosynthesis” and “Linoleic acid metabolism” were the most significant associated pathways according to bioinformatics analysis. In addition, 57 lncRNAs and 36 mRNAs may exhibit significant effects on patient OS rate. Finally, multivariate DEGs in melanoma may have prognostic prediction ability for this disorder.

Emerging evidence indicates that lncRNAs play a vital role in various biological processes such as cell differentiation, immune response, and tumorigenesis (Chen et al., 2018). However, the functions of lncRNAs are not well illustrated (Mercer et al., 2009) and a comprehensive database that provides functions of lncRNAs by experimental researches is still lacking. To further characterize lncRNAs, a “guilt by association” strategy is commonly used to construct a co-expression network of lncRNAs and mRNAs (Liao et al., 2011). In the present study, we used TCGA datasets, which contain evaluated data with high quality and credibility. The transcriptional data was generated by RNA-Seq, which is a revolutionary technology based on next-generation sequencing that is considered the most popular method for studying complete transcriptomes in more detail and with more accurate measurements than microarray and serial analysis of gene expression (SAGE) (Fatica and Bozzoni, 2014). Furthermore, we employed stricter statistical thresholds; fold change > 2 and FDR (*q*-values) < 0.01 were considered significant to obtain DElncRNAs and DEmRNAs. We systematically investigated DElncRNA-DEmRNA co-expression in a series of statistical models using stringent weight above a threshold of 0.8. This approach identified some new DElncRNAs that showed particularly strong co-expression pairs in our results. For example, AC068985.1 to GABRA6, AC093766.1 and AC007953.2 to GCG, MIR202HG to surrounding mRNAs were predicted to exhibit co-expression relationships (weight > 0.99). In addition, some DElncRNAs, such as AL365361.1, AC243960.1, LINC02397, and LINC02422. AL118505.1, LINC01527, AL512274.1, LINC01215, AC012313.4, FAM41C, SSTR5-AS1, were significant both in co-expression network and predicting survival rate, which may show powerful evidence for the metastasis of melanoma. Notably, the majority of the identified lncRNAs were novel, as only a few lncRNAs have been recorded and characterized in literature to date. To our knowledge, our study is the first to identify novel lncRNAs and mRNAs between primary and metastatic melanoma.

Several previous studies have shown that some lncRNAs involved in the pathogenesis of cutaneous melanoma exert their function through different pathways and that they interact with various molecular targets. Sprouty4-intronic transcript 1 (*SPRY4-IT1* or *SPRIGHTLY*), a cytoplasmically enriched lncRNA derived from an intron of the *SPRY4* gene, was the first lncRNA characterized in melanoma in 2011. Khaitan et al. (2011) found that *SPRY4-IT1* modulates apoptosis and promotes melanoma proliferation, invasion, and migration in melanoma cell lines. Subsequent studies showed that *SPRY4-IT1* can function as an anti-apoptotic factor by binding LPIN2 and can regulate lipid metabolism by avoiding cellular lipotoxicity (Mazar et al., 2014). Patients with melanoma and low levels of *SPRY4-IT1* have a longer OS than those with high levels of this lncRNA (Liu et al., 2016). Another study identified the *MIR31HG* lncRNA gene as showing among the greatest expression changes upon BRAF V600E expression. *MIR31HG* expression inversely correlated with *p16INK4A*, and its overexpression may represent a frequent driver event of melanoma progression (Montes et al., 2015). Flockhart et al. (2012) identified BRAF-activated non-coding RNA (*BANCR*) by analyzing BRAF V600E-mutant human primary melanoma specimens. RNAi-dependent knockdown of *BANCR* suppressed melanoma cell migration through upregulation of the chemokine CXCL11. It has also been suggested that melanoma proliferation is promoted by *BANCR*-mediated activation of the ERK1/2 and JNK MAPK pathways (Li et al., 2014). In turn, *GAS5* has heretofore been the only lncRNA reported to act as a tumor suppressor in melanoma. Overexpression of *GAS5* in the SK-MEL-110 melanoma cell line resulted in a decrease in MMP2 expression involving collagen degradation and a reduction in cell migration (Chen et al., 2016). Furthermore, *HOTAIR* (Tang et al., 2013), *MALAT1* (Tian et al., 2014), *UCA1* (Wei et al., 2016), *ANRIL* (Xie et al., 2016), *SAMMSON* (Leucci et al., 2016), *PVT1* (He et al., 2018), and other lncRNAs are involved in the pathogenesis of malignant melanoma; therefore, they appear to comprise promising biomarkers for early tumor detection or disease progression.

Our GO annotation results indicated that DEmRNAs associated with epidermal cell structure and differentiation might contribute to melanoma tumorigenesis. Kodet et al. (2015) have observed that melanoma cells and neural crest increase expression of keratins 8, 14, 19, and vimentin in co-cultured human primary keratinocytes. FGF-2, CXCL-1, IL-8, and VEGF-A also participate in the activity of melanoma cells on keratinocytes. Expression of keratins 7, 8, 14, 16, 18, and 19 was detected by immunohistochemistry in melanoma. MNF-116, keratins 8, and keratins 18 were higher in metastatic melanoma compared with primary melanoma (Safadi et al., 2016).

The most significant pathway identified by KEGG was steroid hormone biosynthesis. Steroid hormones and their various receptor isoforms, such as androgens and estrogens, play an important role in activating and inhibiting the MAPK and PI3K pathways (de Giorgi et al., 2009, 2011; Mitchell et al., 2014). Notably, the incidence of melanoma increased in men at twice the rate that it has been climbing in women since 1975 (NCI, 2012). It may help explain these phenomena to understand the mechanisms by which estrogen and androgen affect benign and malignant melanocytes. In the genomic pathway, unliganded ERα and ARα in the cell cytoplasm can bind estrogen and androgen, respectively, which leads to dimerization of each receptor. ERα and ARα dimers then traffic to the nucleus and bind to protein co-activators such as CREB to alter proto-oncogene transcription and melanoma proliferation. In the non-genomic pathway, the liganded ERα and ARα activate RAS, which activates BRAF, which activates MEK, which activates ERK, also known as MAPK, which can phosphorylate and therefore activate CREB in the genomic pathway or can directly enhance proto-oncogene transcription and melanoma proliferation (Mitkov et al., 2015).

There are several pathways involved in lipid metabolism including linoleic acid metabolism, arachidonic acid metabolism, and alpha-linolenic acid metabolism. Balogh et al. (2010) observed that cell membrane stress achieved either by heat or benzyl alcohol resulted in pronounced and highly specific alterations in lipid metabolism. A phospholipase *C*-diacylglycerol lipase-monoacylglycerol lipase pathway was identified in mouse melanoma B16 cells and significantly contributed to the production of several lipid mediators upon stress including arachidonic acid. Schmitt et al. (2015) found that PRDX6 is strongly expressed in most melanoma cells and its expression levels are maintained in a post-transcriptional manner, particularly by EGFR-dependent signaling, while arachidonic acid acts as a key effector of PRDX6-dependent proliferation and inducer of Src family kinase activation. These results subsequently support the biological importance of the emerging evidence of lipid signaling in melanoma.

Another important signaling pathway in our enrichment results is cytochrome P450. Numerous research groups have shown that individuals with polymorphisms that cause defective CYP2D6 are at increased risk for melanoma (Strange et al., 1999). Other genes such as CYP3A4, CYP2C9, ADH4, ADH1A, and CYP1A2 enriched in the cytochrome P450 pathway are observed as metabolically active procarcinogens to genotoxic intermediates, and an association has been found between CYP enzyme activity and the risk to develop several forms of cancer. Amann et al. (2015) found that the retinoic acid receptor (RAR) regulated enzyme CYP26a1 showed a significantly lower expression in the lecithin retinol acyltransferase (LRAT)-overexpressing murine melanoma B16F10 cell line. The absence of LRAT-catalyzed retinol esterification is important for mediating retinoid sensitivity in murine melanoma cells. The potentially complicated mechanisms of the association between cytochrome P450 and retinol metabolism deserve further studies.

It is noteworthy that a single lncRNA was co-expressed with multiple mRNAs and that multiple lncRNAs could be co-expressed with individual mRNAs. Recent studies have shown that most lncRNAs in humans are produced from divergently transcribed protein-coding genes and that the divergent lncRNA/mRNA pairs exhibit coordinated varies in transcription (Sigova et al., 2013; Cabili et al., 2015), representing cis-acting co-expression of lncRNAs and neighboring mRNAs. LncRNAs may also involve in gene expression by targeting distant (trans-acting) coding genes (Cesana et al., 2011; Chu et al., 2011). We identified both neighboring and distant-acting lncRNA and mRNA co-expression. These phenomena suggest highly complex regulatory relationships between lncRNAs and mRNAs in differential co-expression networks.

This study still has limitations. First, the study power is limited by the lopsided size of the samples. Further researches with lopsided larger sample sizes are warranted to validate the reported findings. However, we took the use of multiple kinds of tests into consideration and applied a strict significance threshold to prevent false positive results. Also, we integrated the expression levels of mRNAs and lncRNAs into co-expression profiles to study their characteristics in primary and metastatic melanoma. Experimental validation of the individual roles of lncRNAs is needed. Furthermore, we classified melanoma into primary and metastatic groups because of limited information, although it is likely that melanoma-associated lncRNAs and lncRNA-mRNA co-expression regulation are specific to more detailed clinical phases as well as histological and molecular subtypes. It is essential to perform subgroup analyses by melanoma subtypes in future studies. Finally, the associations of gene expression levels between lncRNAs and mRNAs such as analyzed in this study may not represent the only possible mechanism by which lncRNAs may function in gene regulation; other mechanisms such as promoter demethylation, chromatin remodeling, microRNA silencing, or serving as molecular scaffolds have been reported (Cesana et al., 2011; Chu et al., 2011).

## Conclusion

Our work reported the novel dysregulated lncRNAs involved in melanoma determined by using bioinformatics analysis, basing on the large scale of clinical data about lncRNAs between primary and metastatic melanoma. These results might serve as the foundation for establishing future diagnostic or prognostic biomarkers and illuminating potential molecular mechanisms in melanoma. Future studies should be conducted to confirm our findings and to elucidate the detailed tumorigenesis mechanisms of the specific identified lncRNAs.

## Author Contributions

LY designed the study, carried out analyses, generated figures, and wrote the manuscript. ZG and SW contributed to statistical assessment, writing, and review. LS designed the study, contributed to writing and review. RT and PL contributed to writing and review.

## Conflict of Interest Statement

The authors declare that the research was conducted in the absence of any commercial or financial relationships that could be construed as a potential conflict of interest.

## Footnotes

1 https://cancergenome.nih.gov/

2 https://string-db.org/

3 https://david.ncifcrf.gov/

4 http://omicslab.genetics.ac.cn/GOEAST/

5 http://www.pantherdb.org/

6 http://www.genome.ad.jp/kegg/

7 https://string-db.org/
